# Expression of somatostatin receptors in canine and feline meningioma

**DOI:** 10.1002/vms3.1537

**Published:** 2024-07-16

**Authors:** Martin Immler, Michael Wolfram, Anna Oevermann, Ingrid Walter, Birgitt Wolfesberger, Alexander Tichy, Gabriele Gradner

**Affiliations:** ^1^ University of Veterinary Medicine Vienna (Vetmeduni), Veterinaerplatz 1 Vienna Austria; ^2^ Division of Neurological Sciences Vetsuisse Faculty, University of Bern, Bremgartenstrasse 109a, 3012 Bern Switzerland

**Keywords:** cat, dog, immunohistochemistry, meningioma, RT‐qPCR, somatostatin receptor

## Abstract

**Objectives:**

The standard treatment for canine and feline meningiomas includes radiotherapy, surgical excision or combined therapy. However, new therapeutic approaches are required due to the possible recurrence or progression of meningiomas despite initial therapy. Adjunctive therapy with synthetic long‐acting somatostatin (SST) analogues has been described in humans with SST‐expressing tumours. The expression of SST receptors (SSTRs) by feline meningiomas is currently unknown, and there are little data about canine meningiomas. We hypothesized that SSTR is expressed by canine and feline meningiomas (S1).

**Methods:**

Seven canines and 11 felines with histologically confirmed meningiomas underwent STTR screening. RNA expressions of SSTR1, SSTR2, SSTR3 and SSTR5 (canine) and SSTR1–SSTR 5 (feline) in fresh frozen and formalin‐fixed and paraffin‐embedded (FFPE) samples were investigated using real‐time (RT)‐qPCR. The expression of SSTR1 and SSTR2 in FFPE samples was evaluated using immunohistochemistry (IHC). The specificity of applied antibodies for canine and feline species was confirmed by western blotting.

**Results:**

In canine meningiomas (*n* = 7), RNA expression of SSTR1, SSTR2 and SSTR5 was detected in all samples; SSTR3 RNA expression was detected in only 33% of samples. In feline meningiomas (*n* = 12), RNA expression of SSTR1, SSTR4, SSTR5 and SSTR2 was detected in 91%, 46%, 46% and 36% of samples, respectively; SSTR3 was not expressed. Overall, the detection rate was lower in FFPE samples. IHC revealed the expression of SSTR1 and SSTR2 in all samples from both species. However, it is important to exercise caution when interpreting IHC results due to the presence of diffuse background staining.

**Conclusions:**

SSTRs are widely expressed in canine and feline meningiomas, thereby encouraging further studies investigating SSTR expression to conduct trials about the effect of adjunctive therapy with long‐acting SST‐analogues.

## Graphical abstract

• Canine and feline meningiomas express somatostatin receptors (SSTRs).

• RNA analysis showed SSTR1, SSTR2 and SSTR5 expressions in canine meningiomas.

• Feline meningiomas exhibited SSTR1, SSTR4, SSTR5 and SSTR2 expressions.

• Immunohistochemistry confirmed SSTR1 and SSTR2 expressions in both species.

• There might be a potential for adjunctive therapy with long‐acting somatostatin analogues, but follow‐up studies with larger groups are required. Potentials of analogues are not evaluated in this study.

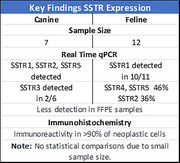

## 1 INTRODUCTION

Meningioma, a slow‐growing tumour of the meninges, is the most common tumour of the central nervous system (CNS) in cats, dogs and humans (Arena et al., 2004; Miller et al., 2019; Troxel et al., 2003). According to the World Health Organization (WHO), it can be categorized into three grades and further histological subtypes (Louis et al., 2007). Meningiomas cause neurological deficits by compressing or invading the brain and other neural structures.

The standard treatments for meningiomas include radiotherapy, surgical excision or a combination of both approaches (Axlund et al., 2002; Goldbrunner et al., 2016). If radiation therapy is declined by the pet owners or surgical removal is not feasible, alternative or adjunctive treatment with long‐acting somatostatin (SST) analogues could be considered. These therapies have already been described for treating human meningiomas if there are signs of recurrence on diagnostic imaging without neurological deficits (Graillon, Romano, Defilles, Saveanu et al., 2017; Wu et al., 2020).

Tetradecapeptide SST inhibits tumour growth by blocking growth factor pathways (Pyronnet et al., 2008). Moreover, its physiological function plays a pivotal role by modulating neurotransmission, secretion and cell proliferation in the pancreas, CNS, gastrointestinal tract and pituitary gland (Patel & Srikant, 1997; Reichlin, 1983). SST also induces apoptosis and cell cycle arrest (Pyronnet et al., 2008).

SST acts by binding to high‐affinity G‐protein‐coupled SST receptors (SSTRs). SSTRs can be divided into five subtypes: SSTR1–SSTR5 (Patel et al., 1995; Weckbecker et al., 2003). SSTR2 is the most widely expressed receptor in human tumours and has the strongest antitumor properties (Weckbecker et al., 2003). The half‐life of SST is usually shorter than 3 min; in contrast, the half‐life of SST‐analogues, such as octreotide, pasireotide and lanreotide, is longer than that of SST, lasting from about 90 min to as long as 4 weeks (Chamberlain et al., 2007; Susini & Buscail, 2006). SST‐analogues were developed for the treatment of neuroendocrine tumours in humans (Godara et al., 2019). The synthetic analogues have a high affinity for SSTR2 and SSTR5, moderate affinity for SSTR1 and low affinity for SSTR3 and SSTR4 (Weckbecker et al., 2003). SST‐analogues have already been used in veterinary medicine for treating feline hypersomatotropism, but not yet in canine and feline meningiomas (Altschul et al., 1997; Gostelow et al., 2017; Scudder et al., 2015).

The presence of SSTRs in human meningiomas has been confirmed by several authors (Arena et al., 2004; Oliveira Silva et al., 2015; Reubi et al., 1986), and SST‐analogues have been applied in the therapy of human meningiomas. A clinical study on human meningioma with long‐acting SST‐analogues reported a progression‐free survival of 6 months in 44% of patients (Chamberlain et al., 2007). SSTRs have been detected in other tissues in veterinary medicine, such as mammary gland tumours, pituitary adenomas and more (Sakai et al., 2015; Scudder et al., 2019). However, only one study has reported SSTR2 expression in canine meningiomas (Foiani et al., 2019). Thus, the data on SSTR expression in canine and feline meningiomas are limited.

This study aimed to investigate the RNA and protein expression of SSTR in canine and feline meningiomas via real‐time (RT)‐qPCR and immunohistochemistry (IHC), respectively. Specific antibody binding for canine and feline tissue was confirmed by western blotting (WB). We also compared whether the manner of sample preparation (fresh frozen or formalin‐fixed and paraffin‐embedded [FFPE] samples) affected the RT‐qPCR results.

## 2 MATERIALS AND METHODS

### 2.1 Data

Tissue samples and data from feline (*n* = 12) and canine (*n* = 7) meningiomas were collected from routine patients who sought treatment at the university veterinary hospital in Vienna from 2007 to 2022. All pet owners signed a declaration of consent for the scientific use of the tumour samples.

The tissue samples were obtained by excision during surgical removal of the tumour or directly after euthanasia. All samples were directly collected and stored by the VetBiobank (Walter et al., 2020). Parts of the samples were frozen immediately after removal or fixed in 4% neutral formaldehyde and embedded in paraffin wax (FFPE). Sex, age, breed, location of the tumour and identification number of the animals were recorded for each meningioma sample (Table 1).

**TABLE 1:** Patient overview and World Health Organization (WHO) grading.

Canine meningioma						
N	Breed	Age (years)	Sex	L	Grade	Subtype
1	GS	10.4	m	Rostrotentorial	I	t
2	Mixed	5.1	fs	Parietal/frontal	I	at
3	Havanese	10.1	fs	SP (T12/13)	I	mt
4	Jack Russell	10.9	fs	Parietal	III	a
5	Maltese	3.7	mc	SP (C1/2)	I	mt
6	Mixed	10	mc	Fossa cranii Cranialis	II	at
7	Labrador	10	m	Lobus olfactorius	I	mt

Feline meningioma						
N	Breed	Age (y)	Sex	L	Grade	Subtype
1	DSH	14	fs	Falx cerebri	I	f
2	DSH	5.1	mc	Parietal	I	t
3	DSH	15	fs	Tentorium cerebelli osseum	I	t
4¶	DSH	14.5	mc	Frontal	I	t
5	BSH	11.8	fs	Parietal	I	f
6	DSH	10.2	mc	Frontal	I	F
7	DSH	11	mc	Lobus temporalis	I	T
8	DSH	11.8	mc	Parietal	I	t
9	DSH	11.5	mc	Parietal/Frontal	I	f
10	DSH	10.0	fs	Frontal	I	t
11¶	DSH	16.8	mc	Frontal	I	f
12	DSH	12.0	fs	Frontal	II	at

*Note*: Summary of the clinical data of the patients presenting with meningiomas in the hospital. The WHO grade and subtype were assessed by a board‐certified pathohistologist.

Abbreviations: a, anaplastic; at, atypical; BSH, British shorthair; DSH, domestic shorthair; EA, extra‐axial; f, fibroblastic; fs, female spayed; GS, German shepherd; L, location; mc, male castrated; mt, meningeal; P, parenchyma; SP, spine; t, transitional (mixed); VBNr, VetBiobank; ¶Same animal number;.

The samples were graded by a board‐certified veterinary pathologist (European College of Veterinary Pathology, ECVP) according to the 2016 WHO classification of meningioma for humans (Louis, Ohgaki et al., 2016, Louis et al., 2021; Louis, Perry et al., 2016). The meningioma was classified as grade III if the mitotic rate was ≧30 mitoses in 2.3758 mm^2^ of tumour surface area, or if histological features of frank anaplasia were present (Yigit et al., 2013). The meningioma was classified as grade II if the mitotic rate was 6–29 mitoses in 2.3758 mm^2^, if brain invasion was present or three of the following criteria were present: increased cellularity, high nuclear/cytoplasmic ratio, prominent nucleoli, uninterrupted pattern‐less or sheet‐like growth and foci of spontaneous necrosis. The meningioma was classified as grade I if the criteria for grade II or III were not met.

### 2.2 RT‐qPCR

#### 2.2.1 RNA of SSTR subtypes 1–5 was quantified using RT‐qPCR

Samples of frozen meningioma tissue of approximately 2 mm^3^ volume were used for RNA extraction. The samples were homogenized using 1.4 mm ceramic beads (VWR) in a MagNa Lyser instrument (Roche) at 6500 rpm for 30 s. RNA extraction was performed subsequently using the RNeasy Mini Kit (QIAGEN) in accordance with the manufacturer's instructions. Genomic DNA was removed using the RNase‐free DNase Set (QIAGEN) in accordance with the manufacturer's instructions.

The RNeasy FFPE Kit (QIAGEN) was used to extract RNA from the FFPE samples in accordance with the manufacturer's instructions. Three to nine 10‐µm FFPE tissue sections were used, depending on the diameter of the embedded tissue. The following steps were equivalent to fresh frozen samples for RNA isolation and DNA digestion.

The RNA concentration was measured using a Spectrophotometer DS‐11 FX+ (DeNovix). The integrity of the RNA was determined using the Agilent 4200 Tape Station System (Agilent Technologies).

cDNA synthesis was performed in accordance with the protocol of the High‐Capacity cDNA Reverse Transcription Kit (Thermo Fisher Scientific Inc), which contained 1 µg total RNA. RT‐minus controls (without enzymes) were included to monitor the amplification of residual DNA. Subsequent to cDNA synthesis, the samples were diluted in 80 µL H_2_O and stored at −20°C.

RT‐qPCR was performed in 20 µL reactions, including 1× HOT FirePol EvaGreen qPCR Mix Plus (ROX) (Solis BioDyne), 200 nM of each primer and 25 ng cDNA.

The samples were analysed in duplicates using the Jena qTower3G qPCR Cycler (Analytik Jena GmbH) in accordance with the following temperature protocol: activation at 95°C for 12 min, 40 cycles at 95°C for 15 s and 60°C for 1 min and termination with a melting curve analysis over a temperature range of 60–95°C.

The following genes were analysed in feline meningioma samples: SSTR1, SSTR2, SSTR3, SSTR4 and SSTR5. Because the SSTR4 gene has not yet been described in dogs, the following genes were analysed in canine meningioma samples: SSTR1, SSTR2, SSTR3 and SSTR5 (Table 2).

**TABLE 2:** Primers that were used in real‐time (RT)‐qPCR.

	Gene symbol	Accession number	Gene name	Oligo	Sequence (5′‐3′)	Amplicon length (bp)	TM
**Canine meningioma samples**	EIF2B1	XM_005636114.2	*Canis lupus familiaris* eukaryotic translation initiation factor 2B subunit alpha	Forward	CCAAGAAGCGTTTCAGTGTG	99	81.5
				Reverse	CAGTGACAGGGACGTTGAG		
	RPL37A	XM_022415249.1	*Canis lupus familiaris* ribosomal protein L37a	Forward	CTCCTGCATGAAGACCGT	104	84.7
				Reverse	ACTGGTCTTTCAACTCCTTCAG		
	SSTR1	^a^XM_005623172.3	*Canis lupus familiaris* somatostatin receptor 1	Forward	ACGGGAGGAGGAAGATGA	1^a^347	84.8
		XM_022421736.1 XM_022421737.1		Reverse	GACAGTCAGGCCGAGTTG	89	
	SSTR2	XM_005623955.3 XM_005623953.3^a^	*Canis lupus familiaris* somatostatin receptor 2	Forward	GCCGAGCTAGCGGATTG	95	82.3
		XM_014116187.2		Reverse	GCCAGCCAGAGATCGTATTC	^a^248	
	SSTR3	XM_005625695.3	*Canis lupus familiaris* somatostatin receptor 3	Forward	GGGCAGCAGAATGATAACCA	123	88.2
				Reverse	CCGATGCAGGATAGCCAAG		
	SSTR5	NM_001286852.1	*Canis lupus familiaris* somatostatin receptor 5	Forward	CTACTTCTTCGTGGTCATCCTG	76	83
				Reverse	GTTGTCAGAGAGGAAGCCATAG		
**Cat meningioma samples**	EIF2B1	XM_006938384.4 XM_006938385.4	*Felis catus* eukaryotic translation initiation factor 2B subunit alpha	Forward	CATCCGGACTTTGCTGGAAT	97	82.7
		XM_006938381.4 XM_006938380.4		Reverse	GCCACACAGGGTTTCTATGG		
		XM_006938382.4 XM_006938383.4					
	RPL37A	XM_004001367.5	*Felis catus* ribosomal protein L37a	Forward	ATAAGCCAGCACGCCAAG	89	83.1
				Reverse	CAGGAACCACAATGCCAGAT		
	SSTR1	XM_019832951.2 XM_019832950.2	*Felis catus* somatostatin receptor 1	Forward	AGGACTTCCAGCCAGAGAA	104	86.5
				Reverse	TGAGGGTCAGGCAGAGTT		
	SSTR2	XM_011289337.3 XM_019816878.2	*Felis catus* somatostatin receptor 2	Forward	GGACTGTGCTACACGACAC	114	82.3
		XM_019816880.2		Reverse	TGTAGTAGACCGCTTCCGT		
	SSTR3_1	XM_019835518.2 XM_019835519.2	*Felis catus* somatostatin receptor 3	Forward	GGCAGCAGGATGATAACCA	102	86.3
				Reverse	CTTTCTCCAGCTCTGACACTC		
	SSTR3_2	XM_019835518.2 XM_019835523.2	*Felis catus* somatostatin receptor 3	Forward	GGCAGCAGGATGATAACCA	78	88.9
		XM_019835519.2		Reverse	GTCAGCGGGCAGCTATT		
	SSTR4	XM_003983838.5	*Felis catus* somatostatin receptor 4	Forward	GAGGCCAAAGGTCAGACAAG	88	86.6
				Reverse	GAGCGCGGTGGAGAGTA		
	SSTR5	XM_006942579.4	*Felis catus* somatostatin receptor 5	Forward	GGTGGTCTTTGCGGACAT	104	88.5
				Reverse	GCACGGAAGTGTAGATGATGAA		

*Note*: A list of primers that were used for RT‐qPCR. EIF2B1 and RPL37A were used as reference genes.

Abbreviations: CLP, *Canis lupus familiaris*; F, forward; FC, *Felis catus*; R, reverse; TM, primer melting temperature.

^a^ Longer amplicon is an isoform that can also be detected with this pair of primers.

EIF2B1 and RPL37A were used as endogenous control transcripts to monitor sample processing (Pfister et al., 2012). The Cq values were determined for all samples in duplicate. Non‐specific signals were removed based on the melting curve analysis.

### 2.3 Western blotting

WB was performed to evaluate the specific binding of the antibodies for canine and feline species.

Portions of positive control samples (brain) were homogenized and immersed in ice‐cold lysis buffer containing 10 mM Tris‐HCl (pH 7.5), 100 mM NaCl, 1 mM ethylenediaminetetraacetic acid, 1 mM ethylene glycol tetraacetic acid, 1% Triton X‐100, 10% glycerol, 0.1% sodium dodecyl sulphate and 0.5% sodium deoxycholate supplemented with 1% (v/v) protease inhibitors (Protease Inhibitor Cocktail; Sigma‐Aldrich). The samples were denatured by heating at 95°C for 8 min. Protein separation was performed on a 10% acrylamide gel (Bio‐Rad Laboratories Inc.). Each lane was loaded with 20 µg of the sample. WB reagent (1:10 in a mixture of Tris‐buffered saline and Tween‐20 [TBST]; Roche Diagnostics) was used to block the membranes. The membranes were probed with SSTR1 (#ASR‐001, Alomone Laboratories) or SSTR2 (#ASR‐002) specific antibodies (diluted in PBS at a dilution of 1:200 and 1:500) and incubated at 4°C overnight.

A secondary antibody conjugated with horseradish peroxidase (1:10,000 in TBST, NA934, Amersham ECL Rabbit IgG, HRP‐linked F(ab′)_2_ fragment from donkey) was applied, and the membrane was incubated for 30 min at room temperature (about 22°C) after adding the secondary antibody. The proteins were visualized using the Bio‐Rad Western Blotting Analysis System (GE Healthcare). The negative control involved the same steps as described above, except that the respective SSTR antibodies were replaced with TBST. Glyceraldehyde‐3‐phosphate dehydrogenase was used as the loading control. To verify the specificity of each antibody, antibodies were preincubated with the respective peptides (BLP‐SR001, BLP‐SR002, Alomone) at 4°C overnight before performing WB.

### 2.4 IHC

IHC was performed for only two receptors due to financial limitations. SSTR2 was used because it has been demonstrated to be expressed in dogs in previous studies and is best stimulated by SST‐analogues (Foiani et al., 2019; Weckbecker et al., 2003). In addition, SSTR1 was chosen based on our qPCR result. Additionally, this receptor was more frequently detected in feline pituitary adenoma, and the respective antibody was available from the same company (Scudder et al., 2015).

Paraffin sections of 2.5‐µm thickness were used for IHC. The slides were incubated in 0.6% H_2_O_2_ in methanol for 20 min at room temperature to block endogenous peroxidase activity. Subsequently, all sections underwent heat epitope retrieval in citrate buffer (0.01 mM) at a pH of 6 in a steamer for 30 min. Protein‐blocking with 1.5% goat serum (Sigma‐Aldrich) in PBS was performed for 30 min to minimize the non‐specific binding of the primary antibody.

The antibodies against SSTR1 (#ASR‐001) and SSTR2 (#ASR‐002) (Alomone Laboratories) were diluted in PBS at a dilution of 1:200 and 1:500, respectively, and incubated at 4°C overnight. BrightVision Poly‐HRP anti‐rabbit (ImmunoLogic) was used as a secondary antibody.

The positive signals were detected using the chromogen DAB (Quanto, Thermo Scientific). The slides were subsequently counterstained with Mayer's Hemalum (Roth), dehydrated and mounted using DPX (Fluka).

The stained slides were scanned using a PANNORAMIC SCAN II (3DHISTECH Ltd.).

To eliminate the potential for non‐specific binding of SSTR antibodies, a pre‐adsorption control was implemented. The same protocol as previously used was followed, with an additional step according to the manufacturer's instructions. The primary antibodies, SSTR1 (#ASR‐001) and SSTR2 (#ASR‐002), were incubated with a blocking peptide for SSTR overnight. For this experiment, the SSTR1 and SSTR2 blocking peptides (#BLP‐SR001 and #BLP‐SR002, respectively) from Alomone Laboratories were utilized.

When the primary antibody was excluded, no signal was detected. However, a weaker signal was still evident when the primary antibody was preincubated with the blocking peptide, in contrast to the slides without. This observation suggests the occurrence of unspecific binding, yet specific binding accounts for the majority of the signal in the analysed tumour samples. For positive control pancreas was used. The pictures were provided in the supplementary material (S1).

**FIGURE 1:**
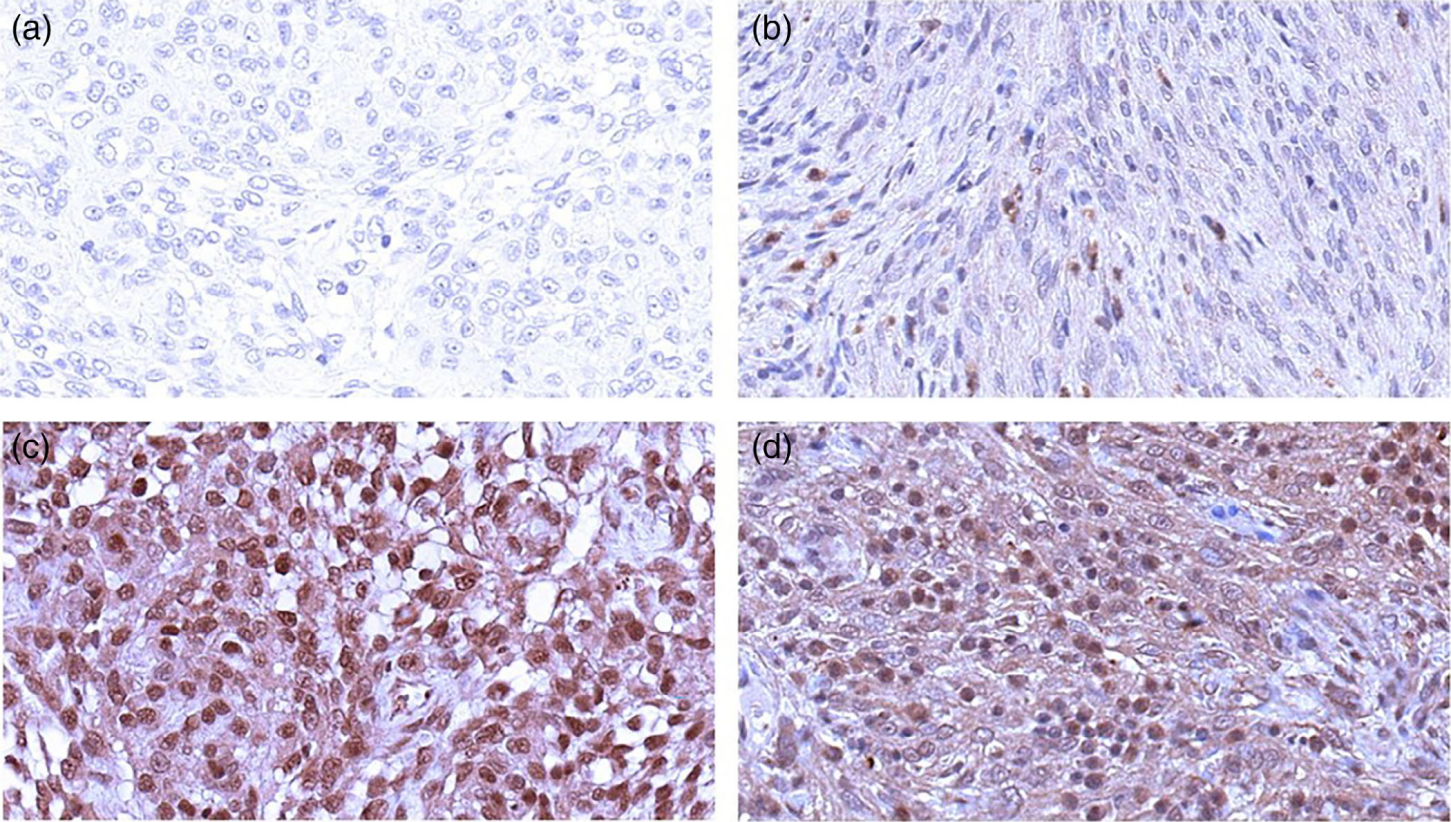
Examples of immunohistochemistry (IHC) scoring for SSTR1. IHC staining examples for SSTR1. (A) serves as the negative control without the secondary antibody addition. (B) represents a feline meningioma with c staining. Letter (C) denotes a feline meningioma with m/c/n staining. (D) signifies a canine meningioma with m/c/n staining. c, cytoplasmic staining; m, membranous staining; n, nuclear staining.

**FIGURE 2:**
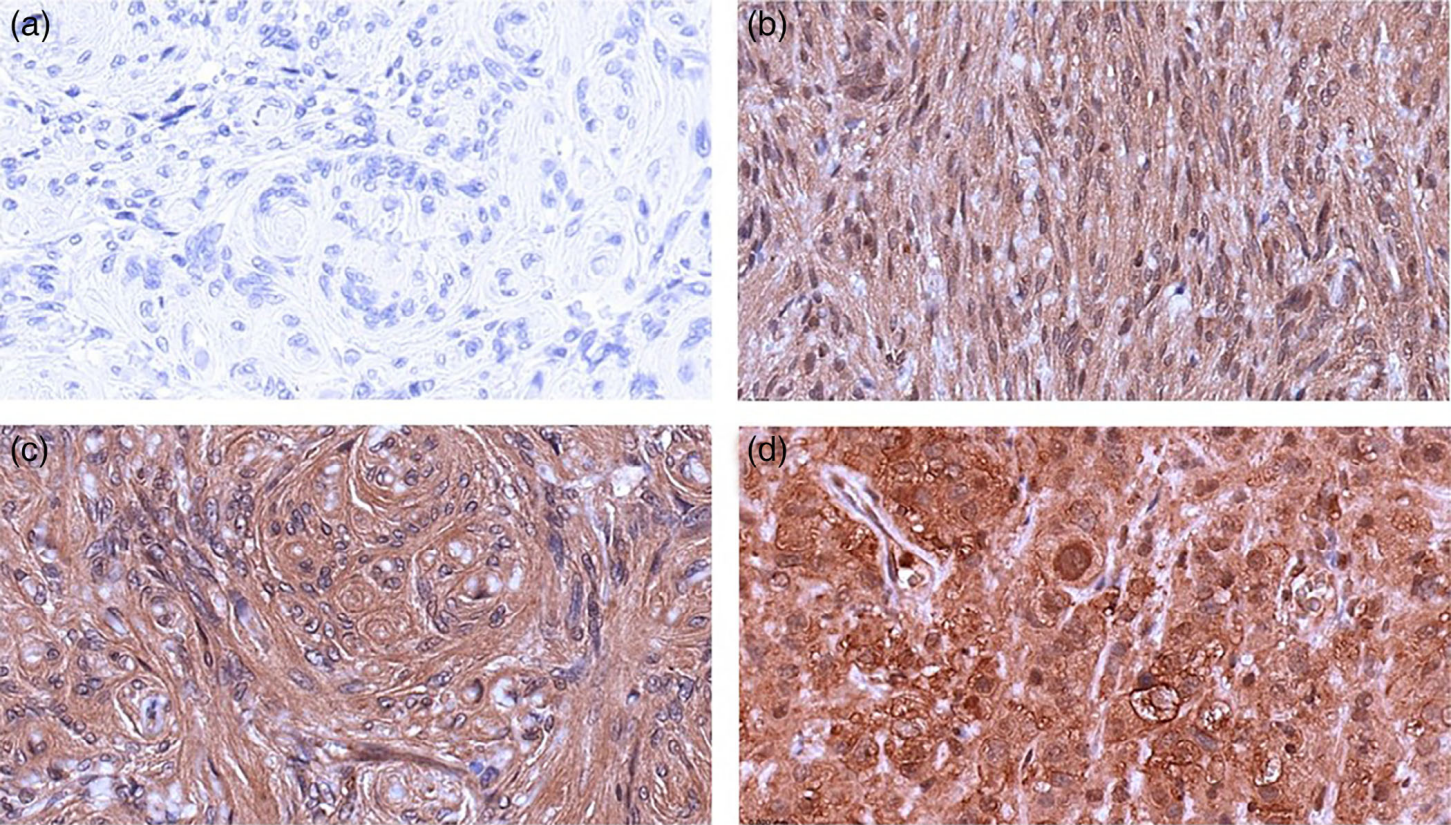
Examples of immunohistochemistry (IHC) scoring for SSTR2. IHC staining examples for SSTR2. (A) serves as the negative control without the secondary antibody addition. (B) represents a feline meningioma with c staining. Letter (C) denotes a feline meningioma with m/c/n staining. (D) signifies a canine meningioma with m/c/n staining. c, cytoplasmic staining; m, membranous staining; n, nuclear staining.

IHC was scored for staining extent and staining pattern. The extent of staining was divided into the following categories: less than 25%, 25%–75% and more than 75%. In addition, the staining pattern (membranous, nuclear and cytoplasmic) was recorded. Figure 1 shows examples of staining patterns of SSTR1, and Figure 2 shows examples for SSTR2.

Non‐specific IgG (Rabbit DA1E mAb IgG XP #3900, Cell Signaling Technology Europe) was diluted in PBS at a ratio of 1:625 and incubated at 4°C overnight to control the non‐specific binding of the detection system and other protein–protein interactions. The same steps as described above were used to detect positive signals using IHC.

### 2.5 Statistics

The data were analysed using IBM SPSS v28. The differences in the detection rates between fresh frozen and FFPE samples were analysed using Fisher's exact test. The differences between the scores of positive and negative samples were analysed using the nonparametric Mann–Whitney test. For all analyses, a *p*‐value below 5% (*p* < 0.05) was considered statistically significant.

## 3 RESULTS

### 3.1 Cases

Nineteen meningioma samples, comprising 12 feline and 7 canine meningiomas, were collected. Two feline meningioma samples were collected from the same cat because the cat had undergone a second surgery due to tumour recurrence 2 years after the first surgery.

The sex distribution of the dogs was as follows: males (*n* = 2); castrated males (*n* = 2), two; and spayed females (*n* = 3). The median age of the dogs was 8.6 years (3.7–10.9 years). The distribution of dog breeds is presented in Table 1.

All six male cats were castrated, and all five females were spayed. The median age of the cats was 12 years (5.1–16.8). Ten cats were domestic shorthair, and one was British shorthair. Twelve feline meningioma samples were removed during surgery, whereas two samples were collected from euthanized cats. Most feline meningiomas were located adjacent to the frontal lobe (5/12), followed by the parietal lobe (3/12). Two canine meningiomas originated from the spinal dura mater. The locations of all the meningiomas are listed in Table 1.

### 3.2 WHO grading

WHO grade I was the most common grade for canine meningioma (5/7), followed by grade II or atypical meningioma (1/7). One dog was diagnosed with grade III or anaplastic meningioma. In the grade I category, 4/7 meningiomas were of the meningothelial and 1/7 of the transitional histotype, respectively.

WHO grade I was the most common grade for feline meningiomas (11/12). One feline meningioma was grade II (atypical). Transitional (50%) and fibroblastic (42%) were the main subtypes of grade I feline tumours (Table 1). The cat that experienced meningioma recurrence displayed WHO grade I in both tumours, but the subtype transformed from transitional to fibroblastic.

### 3.3 Real‐time‐qPCR

RT‐qPCR was performed on seven canine meningioma samples. For one meningioma, no fresh frozen samples were available. Fresh frozen (*n* = 6) and FFPE samples (*n* = 7) were both analysed by RT‐qPCR. SSTR1, SSTR2 and SSTR5 were detected in all fresh frozen samples (6/6), whereas SSTR3 was detected in two of the six samples (Table 3). SSTRs were detected less frequently in the FFPE samples: SSTR1 (5/7) and SSTR2 (3/7).

**TABLE 3:** Real‐time (RT)‐qPCR results.

Canine meningioma											
Fresh frozen						FFPE					
N	SSTR1	SSTR2	SSTR3		SSTR5	N	SSTR1	SSTR2	SSTR3		SSTR5
1	+	+	−		+	1	+	+	−		−
2	+	+	−		+	2	+	+	−		−
3	+	+	−		+	3	−	−	−		−
4	+	+	+		+	4	+	−	−		−
5	+	+	+		+	5	+	−	−		−
6	+	+	−		+	6	+	+	−		−
						7	−	−	−		−

Feline meningioma											
Fresh frozen						FFPE					
N	SSTR1	SSTR2	SSTR3	SSTR4	SSTR5	N	SSTR1	SSTR2	SSTR3	SSTR4	SSTR5
1	−	+	−	−	+	1	−	−	−	−	−
2	+	+	−	+	−	2	−	−	−	−	−
3	+	−	−	+	+	3	+	−	−	−	−
4¶	+	−	−	−	−	4¶	−	−	−	−	−
5	+	+	−	−	−	5	−	−	−	−	−
6	+	−	−	−	+	6	−	−	−	−	−
7	+	+	−	+	−	7	−	−	−	−	−
8	+	−	−	+	−	8	−	−	−	−	−
9	+	−	−	+	−	9	−	−	−	−	−
10	+	−	−	−	+	10	−	−	−	−	−
11¶	+	−	−	−	+	11¶	−	−	−	−	−
						12	−	−	+	+	−

*Note*: An overview of the results of the RT‐qPCR analysis. Compared with that in fresh frozen samples, SSTR1–SSTR5 were detected less frequently in FFPE samples.

Abbreviations: −, not detected; +, detected; ¶, same animal; FFPE, formalin‐fixed and paraffin‐embedded; SSTR, somatostatin receptor.

In cats, 11 fresh frozen and 12 FFPE samples were analysed. SSTR1 was expressed in 91% (10/11) of the fresh frozen samples. SSTR4 and SSTR5 were detected at 46% (5/11) each (Table 3). SSTR2 was detected in 4/11 (36%). For FFPE samples, SSTR1, SSTR3 and SSTR4 could only be detected in different samples each. SSTR2 and SSTR5 could not be detected at all. The recurrent meningioma expressed SSTR1, which the first meningioma also expressed, and additionally expressed SSTR5.

The detection rates of SSTRs to the different histological subtypes of meningioma were not compared statistically due to the small sample size.

### 3.4 Western blotting

**FIGURE 3:**
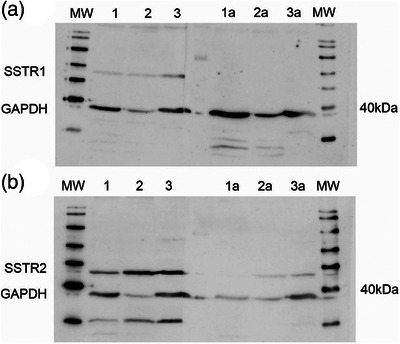
Western blotting (WB). Specificity of the antibodies (SSTR1 [around 60 kDa] and SSTR2 [around 50 kDa]) for canine and feline tissue was assessed using WB. (A) WB for SSTR1; (B) WB for SSTR2. Lane 1, mouse brain; lane 2, dog brain; lane 3, cat brain. Pre‐blocking of the antibodies with the respective peptides before the detection process resulted in a significant reduction or decrease in the specific bands (lanes 1a–3a). Glyceraldehyde‐3‐phosphate dehydrogenase (GAPDH) (42 kDa) was used as the loading control. MW, molecular weight marker.

WBs of respective positive control tissues resulted in bands of the expected molecular weight, and pre‐blocking of the antibodies with the respective peptides before the detection process resulted in a significant reduction in the intensity of the specific bands (Figure 3).

### 3.5 IHC

IHC of 7 canine and 12 feline meningiomas utilizing antibodies against SSTR1 and SSTR2 revealed immunoreactivity in all feline and canine meningiomas. Immunoreactivity was observed in greater than 90% of neoplastic cells. Representative staining results are presented in Figures 1 and 2.

Membranous and cytoplasmic staining were observed most frequently (SSTR1, 43%; and SSTR2, 71%) in canine meningiomas.

In feline meningiomas, membranous and cytoplasmic stainings were most common. The recurrent meningioma exhibited membranous SSTR1 staining, whereas SSTR2 showed additional nuclear staining. The specific stainings of each sample are listed in Table 4.

**TABLE 4:** Immunohistochemistry (IHC) results.

Canine meningioma				
N	SSTR1		SSTR2	
	Staining	Pattern	Staining	Pattern
1	p	m/c/n	p	m/c/n
2	p	m/c/n	p	m/c
3	p	c/n	p	m/c
4	p	m/c	p	m/c
5	p	m/c	p	m
6	p	C	p	m/c
7	p	m/c	p	m/c

Feline meningioma				
N	SSTR1		SSTR2	
	Staining	Pattern	Staining	Pattern
1	p	m/c	p	m/c
2	p	m/c	p	m/c/n
3	p	m/c/n	p	m/c
4¶	p	C	p	m/c
5	p	m/c/n	p	m/c
6	p	m/c	p	m/c
7	p	m/c/n	p	m/c
8	p	c/n	p	m/c/n
9	p	C	p	c
10	p	m/c/n	p	m/c/n
11¶	p	m/c	p	m/c/n
12	p	m/c	p	c

*Note*: Samples were graded according to the presence or absence of IHC staining. IHC staining was present in all cases of feline and canine meningiomas. In addition, the staining pattern (membranous, nucleolar and cytoplasmic staining) was also recorded. Staining extend was in all samples over 75%.

Abbreviations: ¶, same animal; a, absent; c, cytoplasmic; m, membranous; n, nuclear; p, present.

The detection rates for the different subtypes and grades were not compared statistically due to the small sample size.

## 4 DISCUSSION

SSTRs are widely expressed across different tissues in nearly all species. Their potential for anti‐tumour effects can be a crucial treatment for meningioma in dogs and cats. Prior to administering an SSTR‐targeted therapy, one must confirm the presence of SSTR in canine and feline meningioma. Therefore, this study aimed to verify the SSTR expression in meningiomas present in dogs and cats.

SSTR‐targeted therapy is a recommended treatment for inoperable or recurrent meningiomas in humans (Graillon, Romano, Defilles, Saveanu et al., 2017; Chamberlain et al., 2007; Braat et al., 2019; Graillon, Romano, Defilles, Lisbonis et al., 2017; Mirian et al., 2021; Schulz et al., 2011; Seystahl et al., 2016). Octreotide and pasireotide have already been used for the treatment of feline pituitary adenomas and canine gastrinomas, with partial success (Altschul et al., 1997; Gostelow et al., 2017). SSTR‐analogues could also be used as monotherapy or as adjunctive therapy after surgery or radiation therapy to reduce the frequency of recurrence or delay progression. Due to the mild side effects associated with its use, such as diarrhoea and polyphagia in dogs and cats, SSTR‐analogues would be a better therapeutic option for the treatment of weak or older patients, where anaesthesia or surgery may be contraindicated (Khanna et al., 2002; Scudder et al., 2015). According to human data, SSTR‐analogues as monotherapy did not improve the health of the patient due to a lack of tumour shrinkage in cases where neurologic deficits have already been observed (Graillon, Romano, Defilles, Saveanu et al., 2017; Graillon, Romano, Defilles, Lisbonis et al., 2017; García‐Luna et al., 1993; Jaffrain‐Rea et al., 1998).

Several synthetic SST‐analogues have been developed that stimulate the five different receptors to varying degrees (Chalabi et al., 2014). Certain analogues may have a stronger effect, depending on the receptor distribution in the tumour. Pasireotide, octreotide and lanreotide are the most frequently used analogues (Chalabi et al., 2014; Graillon, Romano, Defilles, Lisbonis et al., 2017). Octreotide and lanreotide stimulate SSTR1, SSTR2, SSTR3 and SSTR5, with SSTR2 being the most addressed one. Pasireotide stimulates SSTR1, SSTR2, SSTR3 and SSTR5, with a 30‐ to 40‐fold higher binding affinity to SSTR2 and SSTR5 than octreotide. It is used in the treatment of pituitary adenoma, where SSTR5 is more highly expressed than SSTR2 (Bruns et al., 2002; Chalabi et al., 2014; Godara et al., 2019; Gostelow et al., 2017; Iacovazzo et al., 2016).

In this study, the prevalence of SSTR1–SSTR3 expressions based on PCR analysis in canine meningiomas was 100%, 100%, and 33%, respectively, which was similar to that observed in human meningiomas at 86%–93%, 79%–100% and 43%–60%, respectively (Arena et al., 2004; Dutour et al., 1998). SSTR5 has a higher prevalence in canine meningiomas than in human meningiomas (33%–67%) (Arena et al., 2004; Dutour et al., 1998).

The distribution of SSTR1, SSTR4 and SSTR5 in feline meningiomas was similar to that of human meningiomas (Arena et al., 2004; Dutour et al., 1998). However, the distribution of SSTR2 and SSTR3 was lower in feline meningiomas than in human meningiomas.

The PCR results from our study may be important for choosing the right SST‐analogue in future studies. The discoveries that pasireotide stimulates SSTR1 more than SSTR2 and that it has a 30‐ to 40‐fold higher affinity for SSTR1 than octreotide may allow pasireotide to be used as a treatment option; however, there is currently no clinical data on the treatment effect of pasireotide on dominantly SSTR1 expressing tumours (Bruns et al., 2002; Iacovazzo et al., 2016). Pasireotide also showed a better clinical outcome in cats with SST‐expressing pituitary adenomas (Gostelow et al., 2017; Scudder et al., 2015; Slingerland et al., 2008). However, our study did not specifically investigate the efficacy of different analogues, and no SST‐analogues have been used for the treatment of feline meningiomas.

Only a single study on SSTR2 expression in canine meningiomas has been published; in contrast, no studies on the expression of any SSTR in feline meningiomas are available. However, in the study including canine meningiomas with a detection rate of 56%, only FFPE samples were analysed (Foiani et al., 2019). In our study, the detection rate of SSTR2 was higher in fresh frozen samples than that in FFPE samples, particularly for feline meningiomas; higher degradation of RNA in FFPE material may result in reduced detection of SSTR (Sánchez‐Navarro et al., 2010). Therefore, it is advisable to use fresh material whenever possible when screening for such receptors.

In this investigation, SSTR1 and SSTR2 detection rates in IHC were 100% in meningioma samples from both species. The prevalent, intense and widespread signal was interpreted to represent SSTR expression because preincubation of the primary antibody with the target protein inhibited SSTR detection on tumour slides, and confirmation of antibody cross‐reactivity was obtained through WB. Additionally, IHC data coincided with the PCR data. Furthermore, former investigations have documented similar IHC stain patterns, such as diffuse and cytoplasmic staining (Behling et al., 2022; Sánchez‐Navarro et al., 2010).

However, the interpretation of the IHC results must be approached with caution, as the presence of potential non‐specific antibody binding, as indicated by non‐specific background staining in negative control slides, raises concerns regarding the accuracy of the expression intensity. Therefore, we did not score expression intensity.

Nevertheless, despite these challenges, presenting the IHC results is deemed important as they complement the findings from PCR and WB analyses, providing a comprehensive understanding of the experimental outcomes while acknowledging the limitations inherent in each technique. Further studies should be re‐evaluated using SSTR antibodies from different manufacturers if this issue arises widely and to exclude any non‐specific binding.

Expression rate in canine and feline meningioma was slightly higher than the detection rate in human studies, ranging between 62%–98% and 81%–100% for SSTR1 and SSTR2, respectively (Ahsan et al., 2019; Barresi et al., 2008; Oliveira Silva et al., 2015; Volante et al., 2007). This difference could be due to species specific differences in SSTR expression of meningiomas, different properties of the included meningiomas or the use of different antibodies for IHC. In a study by Foiani et al. (2019), which used the same antibody as us for the screening of 21 canine meningiomas, the detection rate of SSTR2 by IHC was lower (81%), which might be related to the larger number of cases and grade III meningiomas in their study.

Currently, no data are available on the correlation between SSTR1 expression in canine and feline meningioma and therapeutic success. Membranous staining was present in all canine and 83% of feline meningiomas, indicating that SST‐analogue therapy may be a viable option for treating meningiomas as it was found that membrane staining is important for therapeutic success (Volante et al., 2007). However, the assessment scheme of Volante et al. differs from our study. Membranous expression was assessed in our study as well, but it was not divided into fully (grade III) or partially (grade II) expression. Based on the study of Volante et al. (2007), it would be interesting to assess the form of membranous expression in future prospective studies that include the therapy response, particularly if there is a high number of tumours with membranous expression in the retrospective proportion.

Variations in histological subtype, IHC staining and qPCR results were observed between meningiomas obtained from the same cat. It was also noted that histological subtypes can change in human meningioma recurrence (Yamasaki et al., 2000, Arao et al., 1998). In addition, further studies on recurrent meningioma in cats and dogs are necessary to understand the behaviour of these tumours and improve the prediction of recurrence rates based on histological subtype or SSTR expression.

The study is limited by the small sample size and the use of only two out of five IHC antibodies. The sample size depended on the number of surgical cases carried out at the university hospital. Furthermore, due to standardized preparation protocols, only patients whose samples were obtained directly from the VetBiobank were included in the study. Additionally, it is essential to acknowledge the limitations posed by background staining in IHC, which could potentially confound the interpretation of results, especially considering the variability in staining patterns and intensities that may occur with different antibodies. In conclusion, the widespread expression of SSTR in these small cases of canine and feline meningiomas encourages further studies with larger samples to confirm the expression of SSTR. Once expression is confirmed in multiple studies, it will be important to conduct trials of SST‐analogues as an alternative treatment for inoperable meningiomas or in cases where pet owners refuse surgery or radiotherapy. Prior to treatment, SSTR scintigraphy should be performed to determine the patient's suitability for SST‐analogue or peptide receptor radionuclide therapy and, in turn, to determine which SSTR‐analogue will produce the best results. Alternatively, RT‐qPCR of biopsy tissue can determine the expression levels of SSTR1–SSTR5 and guide the selection of appropriate synthetic SSTR‐analogues (Seystahl et al., 2016; Volante et al., 2007; Wu et al., 2020).

## AUTHOR CONTRIBUTIONS

**Martin Immler**: conceptualization (equal); formal analysis (supporting); data curation (equal); investigation (equal); methodology (equal); project administration (lead); validation (equal); visualization (equal); writing – original draft preparation (lead) **Michael Wolfram**: data curation (equal); investigation (equal); Methodology (equal); resources (equal); writing – review and editing (equal); **Anna Oevermann**: data curation (equal); investigation (equal); methodology (equal); supervision (supporting); validation (equal); visualization (equal); writing – review and editing (equal); **Ingrid Walter**: data curation (equal); investigation (equal); methodology (equal); resources (equal); supervision (supporting); validation (equal); visualization (equal); writing – review and editing (equal); **Birgitt Wolfesberger**: supervision (supporting); validation (equal); writing – review and editing (equal); **Alexander Tichy**: formal analysis (lead); writing – review and editing (supporting); **Gabriele Gradner**: conceptualization (equal); methodology (equal); resources (equal); supervision (lead); validation (equal); writing – review and editing (equal).

## CONFLICT OF INTEREST STATEMENT

The authors declare no potential conflicts of interest with respect to the research, authorship and/or publication of this article.

## CELL LINE VALIDATION STATEMENT

There is no cell line validation needed because the samples which were used were tumour samples that were formalin‐ and paraffin‐embedded and fresh frozen samples.

## ETHICS STATEMENT

There was no ethics statement needed because the samples were stored at the institute for pathology.

### PEER REVIEW

The peer review history for this article is available at https://publons.com/publon/10.1002/vms3.1537.

## Supporting information

Supporting Informations

Supporting Informations

Supporting Informations

## Data Availability

The data that support the findings of this study are available from the corresponding author upon reasonable request.
