# Supplementary material for: Expression of somatostatin receptors in canine and feline meningioma
Source: Vet Med Sci. 2024 Jul 16;10(4):e1537. doi: 10.1002/vms3.1537 (PMC11250153; doi:10.1002/vms3.1537)
Supplement: Supplementary file 1 — Supporting Informations [file VMS3-10-e1537-s002.docx]

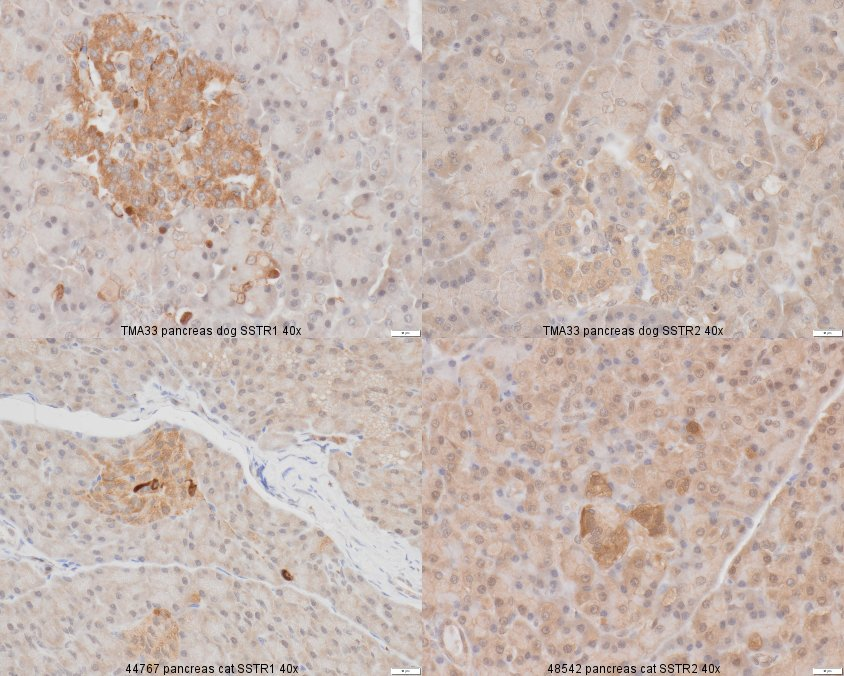

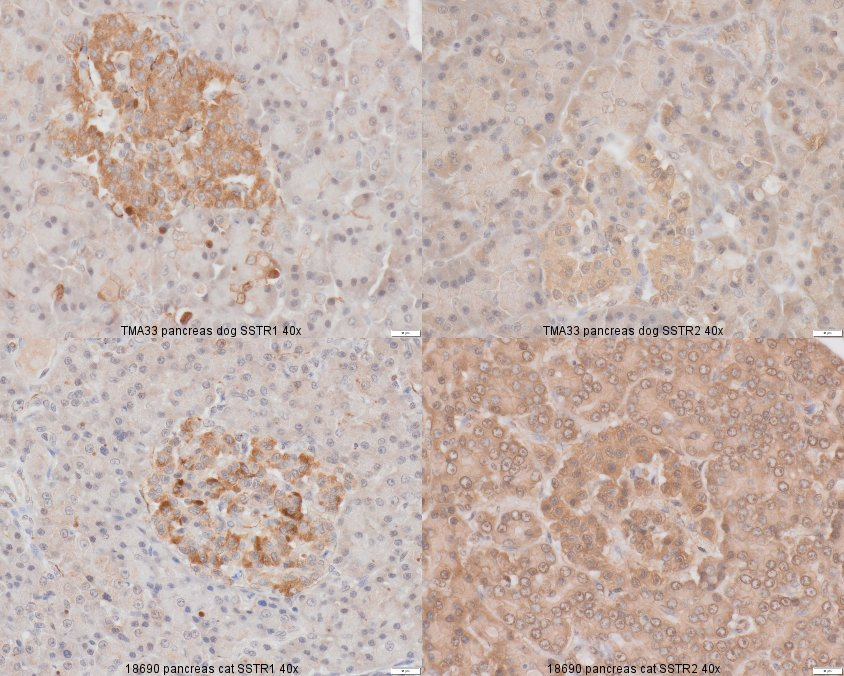


The endocrine pancreas shows convincingly strong SSTR1 signals in sections, while the exocrine pancreas, as expected, shows minor signals in feline and canine tissue. SSTR2 signals in the endocrine pancreas were weaker compared to SSTR1 but still positive, endocrine pancreas showed again some signals as well. This is however comparable to results in human pancreas immunohistochemistry (see <https://www.proteinatlas.org/>).

Doubts have been raised about the specificity and positivity of all the signals observed. Although the controls are convincing, concerns about SSTR1 cannot be entirely eliminated (as is often the case in immunohistochemistry), however, results are supported by PCR and western blot data.
