# Supplementary figures and images for: Expression of somatostatin receptors in canine and feline meningioma
Source: Vet Med Sci. 2024 Jul 16;10(4):e1537. doi: 10.1002/vms3.1537 (PMC11250153; doi:10.1002/vms3.1537)

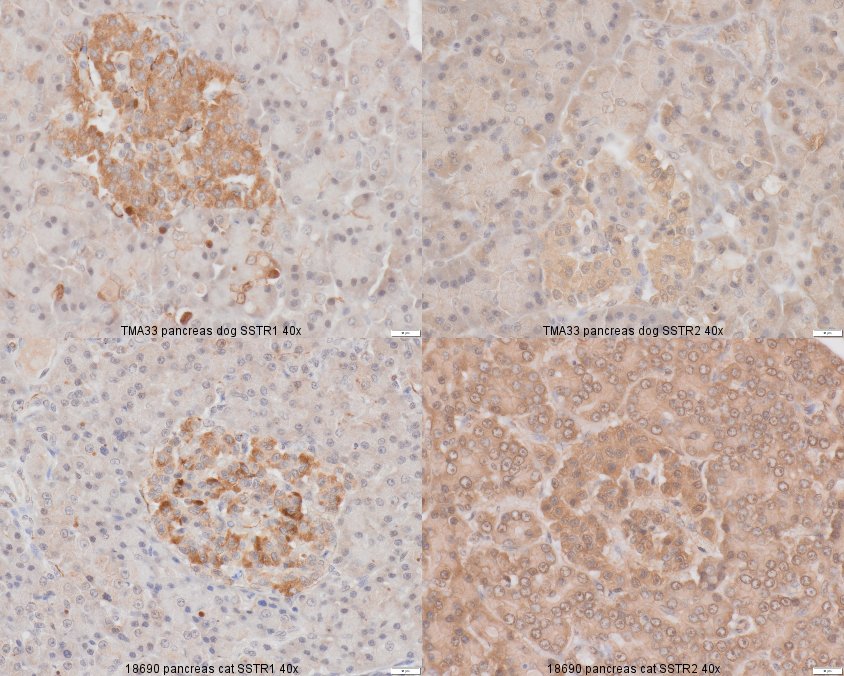

Supplement: Supplementary file 2 — Supporting Informations [file VMS3-10-e1537-s001.jpg]

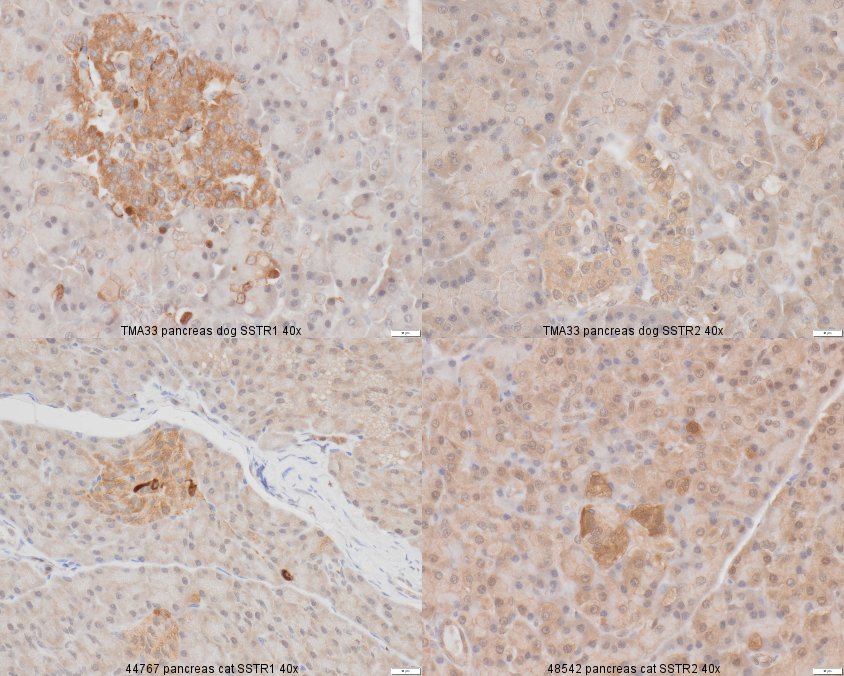

Supplement: Supplementary file 3 — Supporting Informations [file VMS3-10-e1537-s003.jpg]
